# Malaria eradication and elimination: views on how to translate a vision into reality

**DOI:** 10.1186/s12916-015-0384-6

**Published:** 2015-07-25

**Authors:** Marcel Tanner, Brian Greenwood, Christopher J. M. Whitty, Evelyn K. Ansah, Ric N. Price, Arjen M. Dondorp, Lorenz von Seidlein, J. Kevin Baird, James G. Beeson, Freya J.I. Fowkes, Janet Hemingway, Kevin Marsh, Faith Osier

**Affiliations:** 1 grid.416786.a0000000405870574Swiss Tropical & Public Health Institute, 4002 Basel, Switzerland; 2 grid.6612.30000000419370642University of Basel, Basel, Switzerland; 3 grid.8991.9000000040425469XFaculty of Infectious and Tropical Diseases, London School of Hygiene & Tropical Medicine, London, UK; 4 grid.434994.70000000105822706Research and Development Division, Ghana Health Service, Accra, Ghana; 5 grid.1043.6000000012157559XGlobal and Tropical Health Division, Menzies School of Health Research, Charles Darwin University, Darwin, Australia; 6 grid.4991.50000000419368948Centre for Tropical Medicine and Global Health, Nuffield Department of Medicine, University of Oxford, Oxford, UK; 7 grid.10223.320000000419370490Mahidol Oxford Research Unit, Faculty of Tropical Medicine, Mahidol University, Bangkok, Thailand; 8 Eijkman-Oxford Clinical Research Unit, Jalan Diponegoro No.69, Jakarta, 10430, Indonesia; 9 grid.1056.20000000122248486Burnet Institute, 85 Commercial Road, Melbourne, Victoria 3004 Australia; 10 Department of Microbiology, Monash University, 19 Innovation Walk, Victoria, 3800 Australia; 11 grid.1008.9000000012179088XCentre for Epidemiology and Biostatistics, Melbourne School of Population and Global Health, The University of Melbourne, Melbourne, Australia; 12 grid.1002.30000000419367857Department of Epidemiology and Preventive Medicine, Monash University, Melbourne, Australia; 13 grid.1002.30000000419367857Department of Infectious Diseases, Monash University, Melbourne, Australia; 14 grid.48004.380000000419369764Liverpool School of Tropical Medicine, Pembroke Place, Liverpool, L3 5QA UK; 15 grid.463020.30000000121079238African Academy of Sciences, Miotoni Road, Miotoni Lane, House No. 8 Karen, P.O. Box 24916-00502, Nairobi, Kenya; 16 grid.33058.3d0000000101555938KEMRI Centre for Geographic Medicine Research-Coast, Kilifi, Kenya

**Keywords:** Malaria, *Plasmodium falciparum*, *Plasmodium vivax*, Eradication, Epidemiology, Rapid diagnostics, Drug resistance, Mass drug administration, Vaccines, Vector control, Capacity building

## Abstract

Although global efforts in the past decade have halved the number of deaths due to malaria, there are still an estimated 219 million cases of malaria a year, causing more than half a million deaths. In this forum article, we asked experts working in malaria research and control to discuss the ways in which malaria might eventually be eradicated. Their collective views highlight the challenges and opportunities, and explain how multi-factorial and integrated processes could eventually make malaria eradication a reality.

## Introduction

**Fig. 1:**
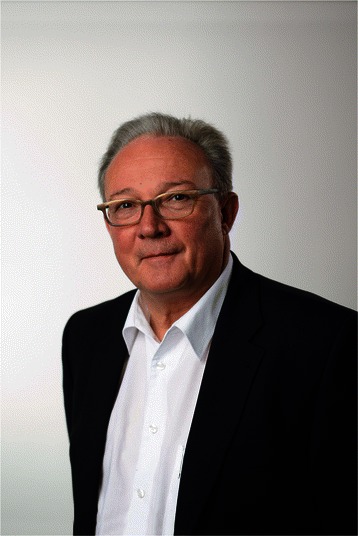
Marcel Tanner is Director emeritus of the Swiss Tropical & Public Health Institute and Professor (chair) of Epidemiology and Medical Parasitology, University of Basel. His research ranges from basic research in cell biology and immunology on malaria, schistosomiasis, trypanosomiasis and filariasis to epidemiological and public health research. Research, teaching and health planning are based on long term work in Africa and Asia

Marcel Tanner (Fig. 1)

Malaria remains the most important parasitic disease, being a major threat in the world and leading to some 600,000 deaths per year. These deaths are predominantly caused by *Plasmodium falciparum* in African children younger than five years old, widespread morbidity, and an under-recognised burden of disease related to *Plasmodium vivax* that puts 2.3 billion people at risk, particularly in Asia [1]. In addition to the ethical reasons, it is also the huge economic burden that calls for global action to reduce and eliminate this intolerable burden for the global benefit. Malaria is a disease of poverty, and malaria control and elimination is a contribution to effective and sustained poverty alleviation.

When the paradigm shift from malaria control to malaria eradication following declarations and plans at the Gates Malaria Forum in October 2007 was re-launched and supported by the World Health Organization (WHO), not only was a new era of slogan-based public and global health action launched but shaken and stimulated by these declarations, scientists and public health actors started to work together in a much more coherent way. They did this by (1) developing integrated control and elimination programmes tailored to a given endemic setting and (2) engaging actively in the research and development (R&D) agenda required for malaria eradication as set by the malERA-process [2]. They also recognised that malaria elimination and subsequently eradication cannot be achieved by the currently existing tools, but require a continuous R&D process for the development of new tools and approaches.

Every year, World Malaria Day forces us to look at where we came from, where we are and what still needs to be done. Joint action over the past decade has led to an impressive impact: malaria infection rates have been cut in half and 4.3 million lives have been saved. Fifty-five countries are on track to reach the World Health Assembly target of a 75 % reduction in their malaria burden by 2015 [3]. Although these huge gains are impressive, they remain fragile if the momentum of the joint action cannot be maintained. Clearly, not keeping the momentum leads to the resurgence of malaria, as we have experienced in numerous previous elimination efforts at national or subnational level (for example, see [4, 5]). It is in this context that the new, jointly established Global Technical Strategy (GTS) by the WHO Global Malaria Programme (GMP) was approved by the World Health Assembly in 2015 [6]. Complementary to this, the follow-up version of the Global Malaria Action Plan [7, 8] by the Roll Back Malaria Partnership (RBM), called Action and Investment to defeat Malaria 2016-2030 (AIM) - for a malaria-free world, will also be launched in 2015 [8]. It is under the umbrella of these two guiding documents that the global health community, leaders and decision-makers together with national programme managers aim at keeping the momentum towards further success and the goals as provided in the WHO/GTS and RBM/AIM (Table 1):

**Table 1:** Joint WHO/GTS (5) and RBM/AIM (7) goals, milestones and targets (adapted from [5])

Goals	Milestones		Targets
	2020	2025	2030
Reduce malaria mortality rates globally compared with 2015	≥40 percent	≥75 percent	≥90 percent
Reduce malaria case incidence globally compared with 2015	≥40 percent	≥75 percent	≥90 percent
Eliminate malaria from countries in which malaria was transmitted in 2015	At least 10 countries	At least 20 countries	At least 35 countries
Prevent re-establishment of malaria in all countries that are malaria-free	Re-establishment prevented		

It is in this spirit that the present Forum, with an impressive set of authoritative view points and analyses, addresses the key areas where our efforts in science and public health actions are still required. First and foremost, it is important that we recognise that the majority of the remaining burden and ongoing transmission is located in the most neglected segments of the populations in endemic areas. More lives could be saved by rigorously increased access to tools, intervention packages and health and social service provisions. These facts suggest that all our interventions, integrated and tailored, within any given health and social system ought to aim at achieving equity effectiveness and not simply cost effectiveness. Besides providing an operational concept and target, equity effectiveness is also the ethical and moral guiding principle to reach the ultimate aim of eradication and an exceptional achievement in the history of mankind with enormous broad societal and developmental benefits for our globe. Consequently follows the motto of the 2015 World Malaria Day reminding us to “*invest in the future – defeat malaria*”.

Clearly, the achievements made so far are remarkable and unprecedented. Looking ahead means now facing the key challenges that remain:

1. The threatening, rapid development and spread of resistance to drugs and insecticides and how we detect, monitor, contain, counteract and possibly eliminate parasites foci from foci where resistance spreads.
2. Maintaining the momentum of drug, vaccine and diagnostics R&D processes that can lead to new tools, which are required to achieve elimination and eradication.
3. Developing and validating effective approaches of mass drug applications for different aims, ranging from cutting transmission to containment of resistance in different population groups and health systems settings.
4. Understanding, developing and coherently implementing health systems and community-based approaches towards surveillance allowing rapid, effective public health action with setting-tailored response packages, i.e. the scientifically grounded operationalisation of the concept of surveillance-response.
5. Guidance through predictive modelling and on the well-synthesised past experience on the effect and costs of combining different interventions and tools in national and subnational elimination efforts, i.e. describing, analysing and modelling case-studies with innovative approaches.
6. The slow progress in better understanding *Plasmodium vivax* and thus understanding the bases for new tools and strategies towards *P.vivax* elimination, a prerequisite for the final aim of eradication.
7. Continuous efforts towards capacity building for scientists, public health specialists and decision-makers to become and remain engaged in the eradication agenda.
8. Assuring continuous, long-term investment in, and funding of, malaria eradication efforts through traditional and novel mechanisms as well as respective domestic allocation.

The contributions in this forum will address these key challenges in more detail.

The WHO/GTS and RBM/AIM have now been adapted to the changing global malaria situation to provide the outlook for action until 2030. However, there still remains the task of carefully and critically refreshing the R&D agenda. It is fortunate that MESA (Malaria Eradication Science Alliance) is currently preparing this crucial update to reach an even more focused and coherent portfolio. This will be tackled, in part, by the public and private sectors, but mainly by various Product Development Partnerships (PDPs) such as Medicines for Malaria Venture (MMV), Innovative Vector Control Consortium (IVCC), Foundation for Innovative Diagnostics (FIND), Novartis Institute for Tropical Diseases (NITD), Drugs for Neglected Diseases Initiatives (DNDi), PATH’s Malaria Vaccine Initiative (MVI) and the European Vaccines Initiative (EVI) that all work in highly pragmatic, efficient processes across and with the private and public sectors. In this respect, we can and must also learn from the successes of the past, particularly the last decade. The most remarkable impact was achieved through a partnership approach that is not only guided by collaborative arrangements but by a true spirit and process of mutual learning for change. Partnership stimulates innovation and public health action, but has also been a main driver of effective capacity building, leadership and health systems strengthening.

Achieving the goals will require major and long-term investments through established and innovative funding schemes, as well as increased and sustained domestic funding of the diseases endemic countries. The current estimates indicate a need of some 6-8 billion USD/year [6] depending on the different milestones (Table 1). Although enormous and extremely challenging for all advocacy and fund raising actions, one should not forget that the return of investment is massive. It was estimated that for each dollar spent, up to 60 USD worth of benefits can be gained for the overall well-being of a society [8–10]. Therefore, advocacy and fund-raising at all levels should be run against this promising background. It is in this spirit that we reflect on World Malaria Day 2015: “Invest in the future – defeat malaria”!

### Competing interests

The author declares he has no competing interests.

## The role of epidemiology in malaria elimination

**Fig. 2:**
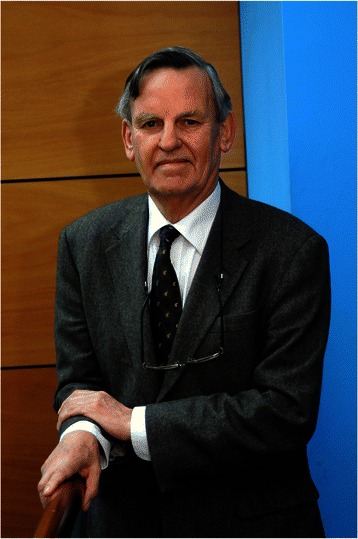
Brian Greenwood is an infectious disease physician who worked and lived in West Africa for 30 years before joining the London School of Hygiene & Tropical Medicine where he is now a professor of tropical medicine. His main research interests are malaria and epidemic meningitis. He has conducted research on many aspects of malaria including its epidemiology, pathogenesis, treatment and prevention and is currently contributing to the evaluation of the leading candidate malaria vaccine RTS,S/AS01

Brian Greenwood (Fig. 2)

Making a commitment to malaria elimination (interruption of all local transmission of the infection in a country or region), especially if a firm time-line is given, is a major step and one which should not be taken lightly. Failure of the first malaria eradication campaign (1955-1969) to achieve its objective led to 30 years of neglect of malaria and this must not be allowed to happen again. A decision to target elimination in a country or region is likely to be influenced by political and financial considerations as well as scientific ones. However, it must be based on a sound knowledge of the epidemiology of malaria in the area that is targeted for attack. Information that is needed includes recent data on the prevalence of malaria infection by age group, the proportion of infections caused by individual malaria species, the identity of the dominant mosquito vectors and their behavior, and knowledge of any social activities in the local population that would put specific groups especially at risk. Information on patterns of drug and insecticide resistance is also required to guide intervention strategies. WHO provides guidelines on the milestones that should be reached before embarking on elimination [11] but these should be considered only as guidelines which may need to be adapted to meet local circumstances.

Recent successes in malaria control have been achieved mainly in areas of previously low transmission in Asia and in regions on the margins of the malaria heartland of central Africa. In many of these areas, elimination of *P. falciparum* malaria is a now realistic goal which is being pursued actively [12], and recently the WHO made a courageous commitment to elimination of falciparum malaria in the Greater Mekong area by 2030 in an attempt to eliminate artemisinin resistant strains of *P. falciparum* [13]*.* Elimination of *P. vivax* will be more difficult than elimination of *P. falciparum* because of the presence of persistent liver stage infections (hypnozoites; the dormant form of the parasite responsible for relapses; this aspect is discussed in a later section of this forum article).

As the incidence of malaria in a particular region declines, transmission usually becomes concentrated in populations who are especially at risk (‘hot pops’) or in geographically restricted areas (‘hot spots’). Examples of ‘hot pops’ are forest workers who camp out in areas where forest vectors reside and artisanal miners who frequently live under appalling conditions. ‘Hot spots’ may be found when the presence of swamps or a persistent water source supports breeding of vector mosquitoes throughout the year. Epidemiological surveillance is needed to detect ‘hot-pops’ and ‘hot-spots’ which can then be targeted for enhanced malaria control [14]. This can be achieved using either active or passive approaches. Establishment of an effective reporting system of cases of clinical malaria, confirmed by microscopy or a rapid diagnostic test (RDT; discussed later in this article), in district health centres, as has been done in Senegal and elsewhere, can provide a rapid means of detecting ‘hot-spots’, provided that the reporting system is accurate and speedy. Active case detection may be needed for ‘hard-to-reach’ populations who do not attend health facilities. Active case detection through formal malaria surveys is demanding and expensive and alternative, less demanding approaches are being explored such as surveys conducted in children attending routine vaccination clinics, women attending ante-natal clinics or school-children.

Until recently, it was widely accepted that in areas of low malaria transmission, nearly all malaria infections caused symptoms which would bring the subject to the clinic. However, it is now apparent that this is not the case and that in some low transmission areas many malaria infections are asymptomatic or cause such minor symptoms that the subject does not seek treatment [15]. Finding these asymptomatic subjects is essential if transmission is to be stopped as they are potentially infectious. Various approaches have been used to do this, including focal screening and treatment of communities considered to be at special risk (FSAT) and mass screening of whole populations (MSAT), but even the latter may miss infected subjects who are away at the time of screening [16]. Detecting asymptomatic infections can be difficult because many are present at only a low density and cannot be detected by conventional RDTs or microscopy. More sensitive tests, such as PCR and the recently developed loop-mediated isothermal amplification (LAMP) assay [17] which does not require a thermocycler, are now being deployed increasingly for this purpose.

If interruption of transmission is achieved, epidemiological surveillance must be sustained to ensure that any introduced infections are detected rapidly and treated. Thus, passive surveillance at hospitals and health centres must be sustained after elimination and regular surveys may be required in population groups or geographical areas known to be at special risk. Finally, in some situations, for example, on islands or in countries with few entry points, screening of visitors from endemic areas can be undertaken to prevent reintroduction of the infection; however, this is difficult to sustain. Studies of the parasite, its vector and its human host are essential in guiding the direction of malaria elimination programmes and in guarding against re-introduction of the infection after success has been achieved.

### Competing interests

The author declares that he has no competing interests.

## Better diagnostic tests for malaria elimination - luxury or necessity?

**Fig. 3:**
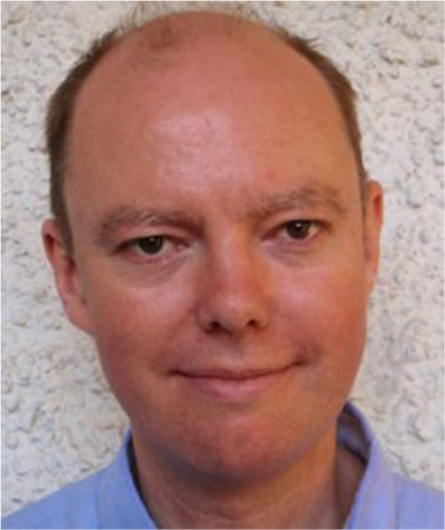
Christopher JM Whitty is Professor of Public and International Health at the London School of Hygiene & Tropical Medicine (LSHTM), and Chief Scientific Advisor and Director of Research at the UK Department for International Development (DFID). He was previously director of the LSHTM Malaria Center and of the ACT Consortium

**Fig. 4:**
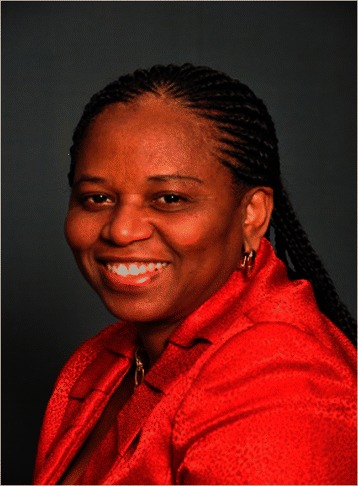
Evelyn Korkor Ansah is a Deputy Director of the Research and Development Division of the Ghana Health Service, an adjunct lecturer at the School of Public Health, University of Ghana and the Chair of the Institutional Review Board of the Dodowa Health Research Center. She is currently one of the two vice-chairs of the Technical Review Panel of the Global Fund and a Steering Committee Member of the ACT Consortium, London School of Hygiene & Tropical Medicine. She has more than 15 years of experience in managing District Health Services including extensive operational research on malaria diagnosis

Christopher JM Whitty (Fig. 3) and Evelyn K Ansah (Fig. 4)

Central to any elimination campaign in its later stages is finding the remaining pockets of transmission, and in its terminal strategies, finding the last few cases. Most diseases targeted for elimination are either easy to spot from their presentation, are chronic, or both; malaria is neither. Initial case-finding is relatively straightforward for smallpox, Guinea worm and polio. Smallpox was sufficiently easy to spot, schoolchildren became case-finders (sensitive) with specific confirmation by experts. Other diseases where eradication is considered, with varying degrees of reality, are chronic (ongoing) making cross-sectional surveys a good way of identifying new cases (e.g. leprosy, filariasis), or involve seroconversion (Yaws). Finding the last cases of malaria is more challenging because it is an acute short-lived disease with non-specific symptoms (fever, headache).

The debate around diagnosis for malaria elimination sometimes implies that the major issue is obtaining more sensitive diagnostic technology. Whilst improved tests may be useful, they are not necessary for malaria elimination in current low transmission settings. In Europe, America and parts of Asia, during the first global eradication campaign, elimination was achieved when only microscopy was available. In areas where malaria transmission has historically been very low it is adequate to identify cases by passive case finding. With a much lower immunity, most infected people will get a fever relatively early in the disease and are likely to present for clinical care. They are diagnosed with current tests, acting as a sentinel that indicates on-going transmission is occurring. This worked for initial malaria elimination in Europe and to identify outbreaks when imported malaria started onward transmission in, for example, Greece or Italy where malaria-transmitting mosquitoes remain [18].

Current rapid diagnostic tests (RDTs) for malaria are both sensitive and specific for clinical malaria [19], simple to use and cost-effective over a range of transmissions including where vivax is predominant [20]. Before trying to design better ones, we need to understand the nature of the new diagnostic challenge [21], and start with the public health problems, not the technical solution. We identify three. The first, for which there is currently no good technical solution, is identifying asymptomatic people with hypnozoites of vivax or ovale malaria (a rare parasite causing a relatively small number of malaria cases) between clinical attacks. Eliminating vivax will be more difficult than falciparum malaria in low transmission settings because of relapse; identifying hypnozoite carriers would be a major advance.

The second is identifying pockets of high transmission during the later pre-terminal phases of an elimination campaign. The initial reduction in transmission at a population level will lead to malaria transmission fragmenting, with hotspots of transmission in a sea of much lower, (eventually no) transmission. Will new technology help here? Arguably, the major problem is that these islands of transmission will be in areas where health services are weak. Identifying incident cases is therefore likely to be difficult for operational reasons. Passive surveillance will need to concentrate on places where marginalised people go for treatment. This is usually the informal private sector where evidence of the impact of improving diagnostics is currently lacking, or peripheral health centres where there is good evidence that RDTs can be used effectively [22]. Providing current diagnostic tools (RDTs) to the shops where patients go and creating incentives for shopkeepers to report may prove much more effective for identifying hotspots than providing improved diagnostic tools to the public sector.

Cross sectional surveys could identify hotspots but given the short-lived nature of malaria infections, they will only work if we have tests which identify recent past as well as current infections. Whilst no serological tests for recent (between two weeks and six months) malaria have proved both sensitive and specific at an individual patient level, at a population level panels of serological markers have proved useful in identifying pockets of high transmission [23]. Combining serology with conventional microscopy in geospatial models has the capacity to identify areas for action in elimination efforts [14].

Once malaria transmission is very low, the third challenge is identifying the few remaining cases. Some argue that more sensitive tests will help this, and certainly they will do no harm. The current problem with over-sensitive tests is that, in high-transmission areas where low level parasite counts are common in asymptomatic people, tests with detection levels below the current cutoff of microscopy will identify large numbers of people with parasites but whose clinical problem is not malaria. More sensitive tests are therefore currently a problem, not a solution, in most of Africa. In any country where elimination is a realistic prospect, it is very unlikely large numbers of people will have low-level asymptomatic parasitaemia, so the disadvantages of more sensitive tests disappear.

We have, in the form of PCR, tests capable of detecting malaria parasites at well below the threshold for microscopy or current RDTs including in pregnancy, an important group for elimination [24]. Operationally, however, PCR is not easy to use outside central laboratories. Developing more sensitive clinical field malaria tests is however possible with current technology.

Whether more sensitive tests will make a significant impact on malaria elimination is less clear. They will not make much difference to passive case detection; by the time people have symptoms, they usually have sufficient parasites to be detected using current tests. The index of suspicion of the person ordering the test for a febrile patient may need to be adjusted, not the test itself, and this involves a complex interaction between patient and healthcare worker [25]. For active case-finding campaigns there is an argument that more sensitive tests will detect people below the level of current parasite detection prior to their getting symptoms. What is not clear is the size of the active case-finding campaign you would have to have in order to make the number of extra malaria cases detected have an appreciable impact on transmission, but it is probably very large. Whether active case-finding on this scale would be practical or cost-effective is uncertain.

Elimination of malaria will require a different approach to malaria diagnosis. Serology-based strategies to identify pockets of ongoing transmission by active case-finding holds promise, but are only as good as the epidemiological sampling used. A near-elimination state takes away the disadvantages of very sensitive clinical malaria tests, but whether more sensitive tests would make a significant difference on transmission is not as clear as is sometimes implied. Much more important for passive case-finding is increasing the likelihood that symptomatic patients present for testing; that public and private healthcare providers test for malaria at a time the disease is rare; and that they report cases. Along with this, there needs to be a systematic active approach to identifying hotspots of transmission. Changing the diagnostic and reporting framework and incentives is, therefore, more likely to have an impact than just improving tests.

### Competing interests

The authors declare they have no competing interests.

## Antimalarial drug resistance undermining malarial elimination

**Fig. 5:**
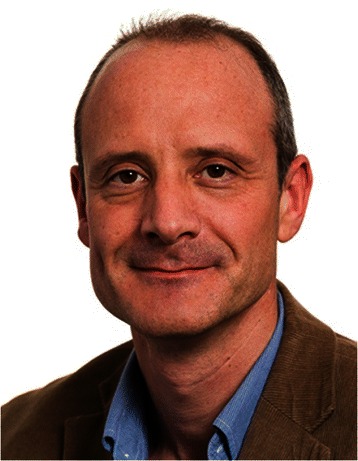
Ric Price is an infectious disease physician, Professor of Tropical Medicine at the University of Oxford, UK and Professor of Global Health at the Menzies School of Health Research, in Darwin, Australia. His research programme focuses on the diagnosis, consequences and containment of multidrug resistant malaria. He is head of the clinical module of the Worldwide Antimalarial Resistance Network (WWARN) and co-Chairs the Vivax Working Group of the Asia Pacific Malaria Elimination Network (APMEN)

Ric N Price (Fig. 5)

Prompt diagnosis and administration of highly effective antimalarial treatment are key components of malaria control programmes of the modern era. Over the last century significant resources have been committed to developing new, safer and more effective antimalarial agents. However, each scientific advance has been followed by the evolution of the parasite and the emergence of drug resistance. As malarial control programmes succeed in reducing the parasite population, the remaining parasites come under increasing selective pressure from intensive drug use. Under such conditions, spontaneous genetic mutations that allow the parasite to survive in increasing concentrations of drug provide a greater chance of survival and onward transmission to a new host [26].

The early clinical manifestations of drug resistance include slower clearance of the parasite biomass and delayed fever resolution. Initially the peripheral parasitaemia may fall below the level of microscopic detection, but if not completely eliminated from the body, subsequent expansion of the parasite population occurs as drug concentrations fall, giving rise to recrudescent infections and clinical representation. Recurrent infections are associated with a greater risk of anaemia and gametocyte carriage. The latter leads to increased transmission of the parasite which can trigger malaria epidemics (Box 1). As resistant parasites become more predominant, recrudescence occurs earlier and the initial parasite clearance takes longer. Eventually high grade resistance results in ineffective treatment, with a greater risk of severe complications and death [27].

**Fig. 6:**
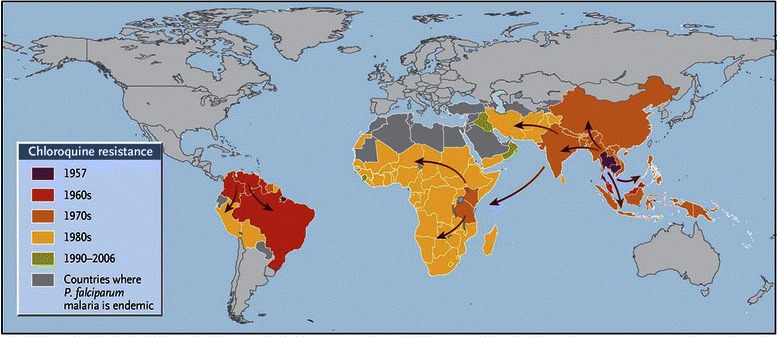
History of chloroquine-resistant *P. falciparum* malaria. Reproduced with permission from Packard, *New England Journal of Medicine* 2014; 371:397-399 [28]. Data are from the WorldWide Antimalarial Resistance Network

Chloroquine played a dominant role in reducing the burden of malaria in the 20th century. However, within 15 years of its initial deployment the first evidence of chloroquine resistance (CQR) began to appear. One of the earliest documentations of CQR came from the gem mines of Pailin in Cambodia. In this area, intense malaria control efforts had included the addition of chloroquine into table salt, thus creating an environment of high exposure of the parasite to sub therapeutic concentrations of drug, an ideal scenario for selection of mutations required for drug resistance [28]. Molecular analyses suggest that the key genetic change in the *pfcrt* gene has arisen spontaneously between five to fifteen times. The resistant parasites then spread along lines of human migration extending the resistance to almost the entire malaria endemic globe (Fig. 6). The subsequent sequential deployment of sulfadoxine-pyrimethamine and mefloquine met with a similar fate; indeed, resistance to these compounds emerged even faster.

A pivotal advance in malaria therapeutics came with the discovery of the antimalarial properties of the Artemisia plant (*Artemisia annua L.* or *A. annua*) [29]. The artemisinin compounds are efficacious even against multi drug resistant strains of malaria, and are associated with excellent tolerability and the ability to reduce gametocyte carriage and thus transmission. However, their rapid metabolism and elimination from the body requires either prolonged treatment courses or combination with longer acting partner drugs. The latter approach has been used to develop artemisinin combination therapies (ACT), which are now deployed as first line treatment in more than 80 malaria endemic countries.

In the last decade, malaria morbidity and mortality have fallen greatly in many endemic areas. Whilst it is hard to quantify the direct contributions to these achievements, it is likely that the deployment of highly effective ACTs has been a critical factor [30]. However, these successes are under threat from the emergence of resistance to the artemisinin derivatives. Early signs of reduced response to artemisinins were first observed in Pailin and the Thai-Cambodian border almost a decade ago. Meticulous prospective clinical studies have defined delayed parasite clearance as the main manifestation of artemisinin resistance [31]. More recently, clinical and molecular studies have demonstrated that delayed parasite clearance times are correlated with mutations in the kelch protein gene on chromosome 13 (*kelch13*) [32], now present throughout mainland Southeast Asia from southern Vietnam to central Myanmar [33, 34]. Reduction in artemisinin efficacy increases the drug exposure of parasite populations to both components of ACTs, facilitating resistance to the longer acting partner drug. The latest victim of such evolutionary pressure is piperaquine, with resistance recently confirmed in patients from an area in Cambodia where artemisinin resistance is greatest [35]. Declining efficacy of these vital combination therapies will eventually reverse the substantial recent gains in malaria control. If resistant parasites spread into the Indian subcontinent and on to Africa, this will have devastating consequences for the most vulnerable populations at greatest risk of malaria.

In areas where the parasite populations are reduced to extremely low levels, the remaining parasites will be the hardest to kill, a phenomenon known as the “last man standing” [26]. When antimalarial drugs are deployed widely, resistance is inevitable, but its emergence and spread can be mitigated by a variety of measures. Ongoing research and development are crucial to identify novel classes of drugs with different modes of action to agents to which relevant mutations have already been selected. The dosing strategy of new drug regimens must be optimised to ensure killing of all parasites, maximising patient adherence to complete a full course of treatment and minimising the exposure of the parasite to subtherapeutic drug concentrations [36]. Poor quality medicines both from substandard manufacturing processes or counterfeit production need to be identified and removed from the market [37]. Vigilance for the emergence and spread of drug resistant parasites and declining treatment efficacy is crucial. Geospatial and temporal mapping of drug resistant data in real time will assist researchers and policy makers to mobilise resources efficiently to contain the resistant parasites early or change treatment practices to more efficacious regimens [34]. The greatest hope for containing newly emerging drug resistant Plasmodia in low endemic settings is to eliminate these parasites at their source by scaling up control efforts [38]. Radical approaches, such as large scale screening and treatment of high risk populations and mass drug distribution, are now being explored with the hope of restricting artemisinin resistance to the greater Mekong region, whilst alternative treatment regimens can be developed. A pharmacopeia rich with antimalarial options and novel strategies to deploy them, will help ensure that malaria control programmes stay one step ahead of the parasite to achieve its ultimate elimination.

### Competing interests

The author declares he has no competing interests.

### Acknowledgements

RNP is a Wellcome Trust Senior Fellow in Clinical Science (091625).

## The role of mass drug administrations in malaria elimination

**Fig. 7:**
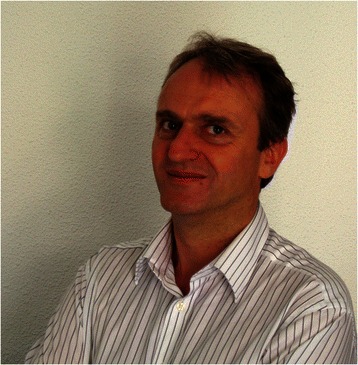
Arjen M Dondorp is a Professor of Tropical Medicine at the University of Oxford, U.K., and a visiting Professor of Clinical Tropical Medicine at Mahidol University in Bangkok, Thailand. He is the Deputy Director and Head of Malaria Research at the Mahidol Oxford Tropical Medicine Research Unit in Bangkok, Thailand. He chairs the Regional Steering Committee for the Global Fund Regional Artemisinin Initiative and chairs the Technical Expert Group on Antimalarial Drug Resistance and Containment for the World Health Organization. His main research interests include antimalarial drug resistance, the pathophysiology and treatment of severe malaria, and care for critically ill patients in resource limited settings

**Fig. 8:**
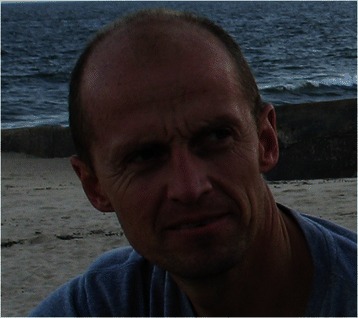
Lorenz von Seidlein has worked for 20 years on malaria and other issues in global health. He worked in The Gambia on the first evaluations of ACTs in sub-Saharan Africa, and has managed several large vaccination projects. He is currently coordinating a major effort to eliminate malaria from areas with artemisinin resistance with the Mahidol Oxford Research unit In Bangkok, Thailand

Arjen M. Dondorp (Fig. 7) and Lorenz von Seidlein (Fig. 8)

The currently applied strategies towards malaria elimination mainly consist of vector control and case management. During the past century stringent implementation of these approaches has eliminated the disease in a number of countries ranging from Australia through the USA and reduced malaria to very low levels in many countries in Asia and Latin America [39]. As long as malaria therapy is efficacious and vector control measures work, continued and concerted efforts should slowly but steadily reduce further the burden of malaria. However, the current emergence of antimalarial and insecticidal resistance threatens to reverse these achievements in malaria control and increases the demand for interventions accelerating malaria elimination.

As outlined in the previous section, parasite populations under antimalarial drug pressure harbouring resistant parasites will select for the most resistant parasites against those drugs, leading to a slow and gradual emergence and spread of resistant malaria, which can go unnoticed for years. The spread of chloroquine resistance through sub-Saharan Africa during the 1980s and 1990s did not attract attention and ultimately resulted in dramatic increases in malaria morbidity and mortality in vulnerable populations [40, 41]. Multidrug resistant malaria became an increasingly urgent and extensive problem in the 1990s, and was only countered with the introduction of a new group of highly potent antimalarials, the artemisinins. In all malaria endemic countries in the world the first line treatment of falciparum malaria is now combination therapy that includes artemisinin derivatives (ACTs). The emergence and spread of artemisinin resistance in the Greater Mekong Subregion in Asia over the last decade [31, 33, 42] initially resulted only in slower clearance of parasitaemia, but now increasingly translates into high treatment failure rates with ACTs because of concomitant partner drug resistance.

Several candidates to replace artemisinin derivatives are in the development pipeline but none is near registration and licensing suggesting that a potential first line antimalarial replacement is many years away [43]. With the current spread of artemisinin and partner drug resistance in Southeast Asia, a resurgence of highly resistant falciparum malaria is a feared and likely scenario. This emergency requires an aggressive response aiming at malaria elimination different from the currently employed control measures.

One such novel approach is the targeted treatment of a defined entire population, such as a village, affected by resistant malaria. The main rationale for this is that this approach addresses the asymptomatic parasite reservoir, which is considered an important contributor to transmission [44]. In addition, the post-treatment prophylactic effect will prevent reinfection of the individual for several weeks, dependent on the pharmacokinetic-dynamic profile of the antimalarial drug. It has become apparent that even in low transmission areas, which is the setting of artemisinin resistance, the asymptomatic parasite reservoir is substantial. Parasite densities are usually low in asymptomatic individuals, and conventional diagnostic tests including microscopy, rapid diagnostic tests, and PCR from filter paper blood spots lack sufficient sensitivity to detect these. Imwong and co-workers have demonstrated that an increase of blood volume used with an ultrasensitive qPCR method results in substantially higher detection rates [45]. The turnaround time of this test is not sufficient to serve a focal or mass screening and treat approach (FSAT or MSAT), whereas more rapid diagnostics currently lack appropriate sensitivity. For this reason, an approach of targeted malaria elimination is currently being trialled where the entire population of a village or other group is treated once malaria prevalence is shown to be substantial using ultrasensitive detection methods. This presumptive antimalarial treatment of targeted populations has been given a range of names including targeted malaria elimination (TME) and targeted malaria treatment (TMT). The premise of this approach is to treat all parasitaemic persons in the population with the aim of malaria elimination. The intervention is designed to be used in a context of well implemented malaria control measures, such as early malaria diagnosis and treatment and vector control.

Mass drug administrations have been successfully deployed against several infectious diseases including lymphatic filariasis. Mass administrations of antimalarial drugs have probably been conducted since antimalarial drugs became available. Perhaps the first documented MDA was done in 1918 [46] and 182 reports of MDAs have since been published, 32 of which complied with the stringent criteria required to be included in a Cochrane Review [47]. The review concluded that “MDA appears to reduce substantially the initial risk of malaria parasitaemia”.

The effectiveness of an MDA depends on the therapeutic efficacy of the drug regimen, the coverage, and the chance of malaria reintroduction from neighbouring endemic areas. The therapeutic efficacy of the intervention depends on the drugs used and the interval of their administration. The drugs should be highly efficacious, persist at therapeutic levels for prolonged periods and should also be be safe and affordable. To minimize the drug pressure it is preferred that the regimen used for MDA differs from the first line treatment in the same area. Current trials (Table 2) use a full course of dihydroartemisinin/piperaquine (DHA-P) and a single low dose of primaquine is added to the drug combination to abort gametocytaemia as quickly as possible [36, 48]. Alternative antimalarials such as artemether combined with lumefantrine can be considered but this partner drug has a shorter half-life and may be more expensive [49]. To treat reinfections due to the survival of infected mosquitoes and re-importation of falciparum malaria by untreated people mathematical modelling indicates that a minimum of three “rounds” of drug administrations is needed to ensure an impact on transmission [50]. Preliminary data show that DHA-P is effective in curing asymptomatic parasite carriers, also in areas with established artemisinin resistance.

**Table 2:** Targeted malaria elimination studies in the greater Mekong Subregion Q2 2015

Country	Start	Drugs	Rounds	Endpoint	Number of villages
Thai Myanmar border Phase 1	Q2 2013	DHA – piperaquine + PQ^a^	3	Parasite prevalence^b^	4
Thai Myanmar border Phase 2	Q4 2014	DHA – piperaquine + PQ^a^	3	Parasite prevalence^b^	300
Vietnam	Q4 2013	DHA – piperaquine + PQ^a^	3	Parasite prevalence^b^	4
Cambodia - Battambang	Q2 2015	DHA - piperaquine	3	Parasite prevalence^b^	4
Cambodia - Preah Vihear	Q2 2015	DHA - piperaquine	3	Parasite prevalence^b^	8
Laos - Savannakhet	Q1 2016	DHA – piperaquine + PQ^a^	3	Parasite prevalence^b^	4
Myanmar	Q1 2015	DHA – piperaquine + PQ^a^	3	Parasite prevalence^b^	8

*PQ* piperaquine

^a^Single low dose primaquine (15 mg)

^b^Parasite prevalence determined by high volume ultra-sensitive qPCR

Ensuring a full treatment course is essential for minimising the risk of additional selection of more resistant parasite populations. With no appropriate alternative antimalarials currently available, there is no real substitute to ACTs for TMT. The potential hazard of increasing drug pressure with ACTs by using a mass drug treatment approach on an already resistant parasite population is well recognised, but should be balanced against its contribution to accelerated malaria elimination in this emergency situation.

The currently available drug regimens have a therapeutic efficacy well above 90 % but even a drug regimen which cures 99 % of the targeted people will only result in 59 % effectiveness if only 60 % of the targeted population participate. Thus, the coverage of the target population is as important as the efficacy of the drug regimen. The key to high coverage is the engagement of the entire target population. Compared with optimising drug regimens, much less is known on how to boost community participation. Each mass drug administration in the past has used a variety of ways to engage and mobilise the target population but the ways and means in which this was done are usually poorly documented and evaluated. Key elements in community engagement are meetings with parts of or the whole target population, house-to-house visits and the use of mass media. Meetings with leaders and key decision makers are critical in the informed consent process as well as in community engagement. In our experience, the most important elements for community engagement are house-to-house visits and face-to-face discussions by trusted community members such as village health workers. Pamphlets and banners may serve as a useful reminder; the effectiveness of mass media such as radio, television and miking (town-criers) is unclear.

The rigorous evaluation of the effectiveness of TMT as a tool for malaria elimination is difficult. Because of the natural fluctuation in transmission intensity between calendar years, a before/after study design is inappropriate, instead a cluster randomised approach is needed to compare intervention with control populations. The unit for intervention is the village, but since villages are highly heterogeneous a large number of villages (“clusters”) would have to be randomised to provide a statistically meaningful interpretation. In addition, reintroduction of malaria from neighbouring areas is a vulnerability of the approach, so that sufficiently large areas will have to be covered for proper evaluation of its potential in elimination.

To identify villages with a high parasite reservoir (hotspots) the village population is tested by taking a venous blood sample. More scalable approaches are needed, for instance testing only a sample of the population with a finger prick blood sample. Current research suggests that within the village, falciparum malaria is transmitted between all demographic strata and geographic locations, so that targeting high risk populations (hotpops) within a village for treatment may not be sufficient.

In the context of the threat of untreatable falciparum malaria in the near future, time for extensive efficacy, effectiveness and implementation studies using conventional trial designs is lacking. Part of the evidence will have to come from a ‘learning by doing’ approach, where moderate scale well defined TMT projects are implemented in low transmission areas with artemisinin resistant falciparum malaria, and coverage, efficacy and safety are carefully recorded. The knowledge and experience thus gained can be used to guide interventions and to model the outcome in other settings.

Elimination of artemisinin resistant malaria implies elimination of all falciparum malaria from the region [26]. In this context targeted presumptive antimalarial treatment of populations could prove to be a pivotal additional tool for malaria elimination. The successful integration of TMT with vector control measures, optimal case management, and perhaps a protective vaccine in the future may well decide whether malaria will be eliminated from many parts of the world.

### Competing interests

The authors declare that they have no competing interests.

## Elimination of *Plasmodium vivax* malaria requires new tools

**Fig. 9:**
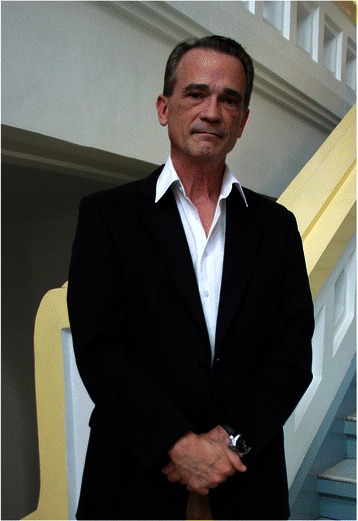
J. Kevin Baird is Professor of Malariology at the Centre for Tropical Medicine, Nuffield Department of Medicine, University of Oxford. He has been Head of Unit at the Eijkman-Oxford Clinical Research Unit within the Eijkman Institute for Molecular Biology in Jakarta, Indonesia since 2007. Kevin serves on several advisory groups and committees for the World Health Organization for the prevention, treatment, and control of *Plasmodium vivax* malaria. He and his Indonesian colleagues conduct laboratory- and hospital-based research and clinical trials of interventions against acute and endemic vivax malaria, especially those aimed at attacking the hypnozoite reservoir of this parasite

J. Kevin Baird (Fig. 9)

Transmission of *P. vivax* occurs all across the endemic tropics and extends into temperate zones such as the Korean Peninsula [1]. A dormant stage of *P.vivax*, the hypnozoite, separates this species from *P. falciparum* in crucially important respects. Where both species occur, *P. vivax* proves much more resilient in the face of conventional methods of control and elimination. Those tools – diagnosis, treatment of the acute attack, and interventions against the mosquito vector – have almost no impact on the hypnozoite. Infection by the hypnozoite is silent and no technology can diagnose it, and the mosquito has no role in attack stemming from the hypnozoite reservoir resident in human communities.

In most endemic settings the hypnozoite reservoir cannot be attacked due to inadequacy of the only drug effective at doing so, primaquine. That problem is not poor efficacy but exceedingly poor effectiveness driven by a specific problem of toxicity. Administering primaquine in relatively small daily doses over 14 days mitigates the serious threat it poses to patients having an inborn deficiency of glucose-6-phosphate dehydrogenase (G6PDd). That condition is the most prevalent and diverse inherited human disorder, affecting over 400 million people, most of them resident where malaria is endemic [51]. This deceptively simple problem denies patients access to primaquine therapy, be they G6PD normal or deficient, because the diagnosis of G6PDd has been beyond the reach of patients at the periphery of healthcare delivery in the endemic rural tropics [52]. The blind administration of primaquine where close clinical supervision cannot occur risks serious harm, but withholding the treatment also does so. The threat of hypnozoite infection without primaquine therapy, i.e., maturing to acute clinical attacks called relapses, is very serious.

The likelihood, timing, and frequency of relapse in *P. vivax* vary by region [53]. In Southeast Asia most strains of *P. vivax* behave like the Chesson strain from New Guinea. Relapses occur in almost all infections and do so rapidly at about two-month intervals. Five or more relapses may be typical for these strains, and as many as twenty attacks in two years have been documented. The incidence density of first relapse in groups of patients not given primaquine approaches five per person year [54, 55]. Among patients diagnosed and treated for acute *P. falciparum* malaria in relatively low transmission areas of Thailand, 50 % experienced an attack of *P. vivax* by relapse within just two months [54]. That figure gives a glimpse of the prevalence of hypnozoite infection in endemic areas.

Despite long being regarded as benign, acute vivax malaria often takes a pernicious course with consequences including severe anaemia, respiratory distress, liver and kidney dysfunction, seizures and coma, haemorrhage, and circulatory collapse [56]. In hospital-based studies of patients suffering these complications, the risk of death closely approximated that of patients suffering the same caused by *P. falciparum* [56]. Patients lacking access to primaquine therapy suffer repeated clinical attacks with attendant risk of severe illness and death, in addition to onward transmission of the infection. The hypnozoite reservoir of *P. vivax* seriously threatens patients and communities.

Eliminating *P. vivax* transmission requires attacking the hypnozoite reservoir. Primaquine is the only means of doing so, and haemolytic toxicity drives its inadequacy for this task. The average prevalence of G6PDd in malaria endemic countries is 8 % [51], but the difficulty of identifying that minority also denies the G6PD-normal majority the enormous clinical and public health benefits of primaquine therapy. In non-pregnant, G6PD-normal patients, primaquine is an extraordinarily safe and well-tolerated drug with superb efficacy despite six decades of continuous use [54, 57]. Providing those patients routine access to primaquine represents a crucial objective in reaching for the elimination of vivax malaria. Achieving it will require rolling out point-of-care diagnostic devices to the periphery of healthcare delivery [58].

A promising new drug against hypnozoites, tafenoquine, is approaching availability [59]. Tafenoquine offers the enormous advantage of good efficacy against relapse with just a single dose, but it also suffers the problem of haemolytic toxicity among G6PDd patients. The great promise of tafenoquine emphasises the urgency of solving the problem of G6PD diagnosis that would otherwise deny most patients access to the therapy.

Solving the G6PD diagnosis problem will nonetheless leave many patients without treatment against relapse. In addition to those found G6PD deficient, pregnant women and infants also cannot receive primaquine, as will likely be the case with tafenoquine. Further, very recent studies suggest relatively common alleles of the P450 cytochrome that metabolises primaquine to its active form (2D6 isoenzyme) render the treatment wholly or partially ineffective against relapse [60]. Strategies for coping with the threat of relapse in these patients have not been explored and will require the hard work of being conceived, evaluated, optimised, and validated prior to any broad implementation. This will be especially important for pregnant women and their foetuses and infants, as they are particularly vulnerable to life threatening complications associated with acute vivax malaria [61].

The prospects for eliminating endemic *P. vivax* will be very bright with the development and roll out of a relatively modest suite of new tools: robust G6PD diagnostics; a single dose therapy against relapse; and strategies for managing patients lacking access to that treatment. Putting these tools into the hands of the providers of care for malaria patients would greatly accelerate the elimination of this species. Doing so requires deliberate effort in research and practical implementation on a global scale.

### Competing interests

The author declares that he has no competing interests.

### Acknowledgements

JKB is supported by Wellcome Trust grant B9RJIXO.

## Progress towards vaccines for the prevention and elimination of malaria

**Fig. 10:**
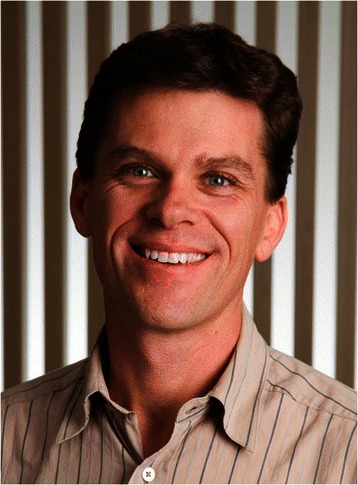
James Beeson is Head of the Centre for Biomedical Research at the Burnet Institute, Australia, and leads a research group focused on human immunity and vaccines against malaria, including clinical and population studies of malaria in Africa, Asia and Papua New Guinea. James trained in clinical medicine and public health, and completed a PhD on malaria in pregnancy at the University of Melbourne, Australia and the Walter and Eliza Hall Institute, Australia

**Fig. 11:**
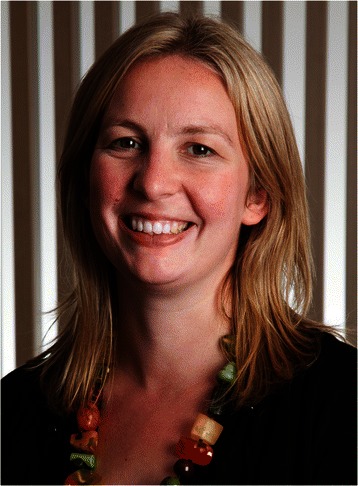
Freya Fowkes is Head of the Malaria and Infectious Disease Epidemiology group at the Burnet Institute, Australia. She trained in parasitology at the University of Glasgow, UK, and then obtained an MSc in epidemiology from the London School of Hygiene and Tropical Medicine, UK, before completing her doctorate in infectious disease epidemiology at the University of Oxford, UK. Freya is involved primarily in examining the epidemiology of malaria, in particular drug resistance, immunology, host genetics and susceptibility to malaria and associated morbidities

James G Beeson (Fig. 10) and Freya J.I. Fowkes (Fig. 11)

The need for an effective malaria vaccine to combat the high global burden of malaria and achieve the long term goal of elimination and eradication is paramount, particularly in the era of emerging resistance to artemisinins and vector control interventions. The broad objectives of malaria vaccines are to reduce morbidity and mortality and reduce the prevalence of infection in populations and interrupt malaria transmission. Vaccines are generally classified into three approaches: pre-erythrocytic vaccines aim to prevent blood-stage infection; blood-stage vaccines aim to clear parasitaemia and prevent clinical disease; and, transmission-blocking vaccines to prevent infection of mosquitoes and interrupt malaria transmission in populations. There are merits in each approach (Table 3), but there is a growing appreciation that vaccines combining multiple targets and stages will be required for achieving and sustaining elimination. Vaccine development has largely focused on pre-erthrocytic and blood-stage vaccines of *P. falciparum* with the focus of preventing morbidity and mortality. However, the potential of transmission-blocking vaccines in malaria elimination is increasingly being recognised, as is the need for *P. vivax* vaccines.

**Table 3:** The role for different vaccine approaches in preventing malaria infection, disease, and transmission

Vaccine objective	Vaccine targets				
	Pre-erythrocytic	Blood-stage	Gametocytes and mosquito stages	Combined Pre-E and BSV	Combined: All stages
Protection against infection	++^a^	+^b^	−^c^	+++	+++
Protection against disease	++	++	-	+++	+++
Reduce transmission	++	?^d^	++	++	+++

Legend to scoring : +, weak effect; ++ modest effect; +++, strong effect

^a^Pre-erythrocytic vaccines have shown significant efficacy against symptomatic malaria and infection, but it has proved to be difficult to achieve a strong degree of protection against infection

^b^Blood-stage vaccines primarily aim to prevent clinical illness and have generally only been weakly protective, on their own, against infection per se

^c^Transmission-blocking vaccines do not directly protect individuals from infection or disease

^d^Blood-stage vaccines may reduce transmission because they reduce parasite density [80], but this remains to be quantified

Abbreviations: *Pre-E* pre-erythrocytic, *BSV* blood-stage vaccines

RTS,S is the most advanced vaccine candidate and targets the pre-erythrocytic stage of *P. falciparum*. RTS,S consists of a circumsporozoite protein construct fused to hepatitis B surface antigen [62] formulated with a new adjuvant, AS01 [63]. Vaccine efficacy of RTS,S (per-protocol) in various Phase II trials in Africa, where the primary endpoint was first or only clinical episode, was modest ranging from 30-66 % in infants and young children [64–68]. In 2009, a large multicentre Phase III trial of RTS,S/AS01 at 11 sites in 7 sub-Saharan African countries, involving 15,460 children (three vaccine doses) was initiated. Efficacy at 12 months in children 5- to 17-months old was 56 % and 47 % for clinical and severe malaria respectively [69] and was lower in infants 6- to 12-weeks old when administered in conjunction with Expanded Programme on Immunisation (EPI) vaccines (31 % and 37 %, respectively) [70].

Waning vaccine efficacy was noted in Phase IIb trials [66, 71, 72] and within 18 months of surveillance in Phase III trials [69, 70, 73]. How immunity wanes overtime is unclear, but RTS,S immunity may be mediated by both vaccine-induced antibodies and cellular immune responses [72, 74–78]; however, there is currently no immune correlate to serve as a strong surrogate of vaccine efficacy to assess longevity of vaccine efficacy. Other factors influencing efficacy are poorly understood. In Phase III studies, there was variation in efficacy between study sites, but it could not be clearly attributed to differences in malaria transmission [73].

Data have recently been released on the efficacy of RTS,S over extended follow-up (three to four years) and on the effect of a booster dose at 20 months [79]. Overall, there was significant vaccine efficacy, and evidence of better efficacy in those who received a booster. Importantly, children who did not receive a booster dose had no significant efficacy against severe malaria. In children 5- to 17-months old at enrolment, vaccine efficacy over four years was 36 % (32 % for severe malaria episodes) in those who received a booster, and 28 % in those who did not (no efficacy against severe malaria). In infants 6- to 12-weeks old followed for three years, efficacy was 26 % (18 % for severe malaria) in those who received a booster, and 18 % (10 % for severe malaria) in those who did not. There was evidence of waning vaccine efficacy during the follow-up period. The vaccine is currently being evaluated by the European Medicines Agency; depending on the outcome, the World Health Organization may make the first malaria vaccine policy recommendations in late 2015.

A small number of other *P. falciparum* vaccine candidates have progressed to phase II trials [80, 81]. However, achieving significant efficacy or potent and sustained anti-malarial responses has been challenging. Vaccines based on pre-erythrocytic targets (ME-TRAP), inducing T-cell effector responses, and delivered using prime-boost strategies showed promise in animal models and human infection challenge models, but failed to show any efficacy in phase II trials in African adults and children [82]; different delivery platforms and regimens are currently being investigated [83]. For blood-stage vaccines, Phase I/II trials evaluating merozoite surface protein (MSP)-2 [84], and apical membrane antigen 1 (AMA1) [85] showed some strain-specific efficacy against malaria. These trials highlight an additional challenge; that of antigenic diversity of candidates and the need for strategies to overcome vaccine escape [86–88]. Follow-up data from a MSP3 phase I vaccine trial suggested significant efficacy [89]. Together, these data provide promise that merozoite targets could form the basis of effective vaccines, alone or in combination with pre-erthrocytic targets, but also highlight the difficulties in developing highly efficacious vaccines against malaria. Several other vaccines have progressed to phase 1 trials. These include merozoite targets (MSP2 bi-allelic vaccine, multi-allelic AMA1, MSP3-GLURP, EBA175, SERA5), pre-erythrocytic antigens (CSP, CelTOS, LSA1) and transmission-blocking candidates (Pfs25), as well as multi-stage combinations [81]. Recent studies are revealing other attractive vaccine candidates, such as EBA and PfRh invasion ligands that play key roles in host cell invasion [90] and are targets of human immunity [91, 92].

Transmission-blocking vaccines generally aim to induce antibodies that will block mosquito infection. A leading candidate is Pfs25, an antigen expressed by ookinetes in the mosquito midgut; Phase I trials report the induction of antibodies that block transmission to mosquitoes in the laboratory [93]. Future requirements are induction of more potent responses and demonstration of transmission-blocking activity under field conditions, as well as evaluation of other transmission-blocking targets such as Pfs230 and Pfs48/45 [94, 95]. An alternate approach is to use whole attenuated *P. falciparum*. Repeated intra-venous inoculations with radiation-attenuated sporozoites were recently shown to give a high level of efficacy against experimental human infection [96]. Challenges include addressing storage, delivery, and administration routes for possible future implementation.

Currently only three *P. vivax* vaccine candidates (*Pv*DBP, *Pv*CSP and Pvs25) have reached clinical trials (Phase I) [97–99]); at present, no phase II field trials of *P. vivax* vaccines have been published. This may reflect the previous neglect of *P. vivax*, and technical challenges such as maintaining *P. vivax* in culture, and limited animal models of infection. *P. vivax* Duffy-binding protein (*Pv*DBP) is a leading vaccine candidate because *P. vivax* invasion of erythrocytes is largely dependent upon its interaction with the Duffy blood-group antigen [100]. *Pv*DBP induces antibody responses in populations naturally exposed to *P. vivax* [101] which may protect against high density *P. vivax* infections [102]. The success of RTS,S, based on *Pf*CSP, suggests that vaccines based on *Pv*CSP may be an appropriate strategy. *Pv*CSP vaccines have been tested in immunogenicity trials [97, 98], but not yet in phase II field trials. The *Pv*s25 transmission blocking vaccine candidate generated antibodies able to inhibit parasite development in mosquitoes in a phase 1 trial, but levels were considered too low for an effective vaccine [99]. Research into other *P. vivax* immune targets has largely been focused on orthologues of *P. falciparum* that elicit antibody responses including *Pv*AMA1, several *Pv*MSPs *(Pv*MSP1, *Pv*MSP3α, *Pv*MSP-5 and *Pv*MSP-9) and *P. vivax* reticulocyte binding proteins (PvRBP1 and PvRBP2) [101]. Much more research is needed to identify and prioritise lead *P. vivax* candidates from pre-clinical studies into clinical trials.

The increasing emphasis on achieving elimination of malaria from numerous regions, and ultimately global eradication, highlights the need for strongly efficacious vaccines that protect against clinical disease and infection, and also prevent ongoing transmission in populations (Table 4). To achieve this will almost certainly require multi-component vaccines that include multiple antigens from different life stages. The challenges in achieving highly efficacious vaccines with single antigen approaches also suggest that multi-antigen and multi-stage vaccines will be required. Vaccines for the Asia and Pacific regions would ideally protect against *P. falciparum* and *P. vivax*. Vaccines with sustained efficacy over several years would also have tremendous benefit. To facilitate elimination, vaccines will need to be implemented in co-ordination with other malaria control interventions. This may lead to synergistic effects for malaria control, but will also maximise the use of limited resources that are typical in malaria-endemic regions.

**Table 4:** Research priorities for the development of vaccines for malaria elimination^a^

**Knowledge of human immunity**
Identification of correlates of immunity for pre-erythrocytic, blood-stage, and transmission-blocking vaccine candidates^b^
Mechanisms of immunity: protection against infection and disease, and transmission-blocking
How long-lasting immune responses and immunological memory are generated through natural exposure
Influence of existing naturally-acquired immunity on responses to vaccines and vaccine efficacy^c^
Quantify the significance of antigenic diversity and the potential for vaccine escape
**Vaccine antigens and combinations**
Developing CSP-based vaccines, or other pre-erythrocytic vaccine candidates, for greater efficacy against infection^d^
Identification and prioritisation of blood-stage and transmission-blocking vaccine candidates for inclusion in combination vaccines
Antigen combinations that induce high levels of immunity against infection and disease, and strong transmission-blocking activity^a^
Combined *P. falciparum* and *P. vivax* vaccines
**Vaccine approaches and technologies**
Vaccine strategies or approaches to induce long-lasting immunity^e^
Adjuvants and delivery systems for induction of potent immune responses
Antigen expression platforms optimised for production of multi-antigen vaccines^f^
Vaccine technologies to simplify the implementation of mass vaccination (e.g. needle-free systems, reduced cold-chain requirements)
Integration of malaria antigens into existing childhood vaccines^g^
**Other**
Tools to monitor vaccine coverage and predict protection
Understand the potential efficacy/impact of vaccines in different transmission settings

^a^The broad aims for malaria elimination vaccines would be vaccines with a high level (>80 %) of efficacy against malaria infection and disease, and a strong potential to reduce malaria transmission by preventing infection or blocking transmission, or a combination of both.

^b^Correlates of immunity would greatly facilitate prioritisation of antigens and combinations for vaccine development, and aid the evaluation of vaccines in clinical trials

^c^There is some evidence that pre-existing naturally-acquired immunity influences the protective efficacy of the RTS,S vaccine.

^d^The RTS,S vaccine has established the potential of vaccines based on the circumsporozoite protein (CSP), but efficacy may be improved by different constructs, vaccine formulations, or additional antigens

^e^The longevity of immune responses reported for RTS,S and other malaria vaccines is shorter than many other licensed vaccines for other pathogens

^f^A single platform for the production of vaccine antigens would be an advantage for achieving highly efficacious multi-antigen malaria vaccines

^g^While malaria vaccines could be given concurrently within the childhood EPI programme, the inclusion of malaria antigens into existing childhood vaccines, to be administered as a single product, would facilitate mass administration and simplify EPI regimens

While the efficacy of RTS,S is modest, and the future effectiveness of RTS,S is yet to be established, the number of clinical cases averted by its implementation is likely to be considerable given the global burden of malaria. RTS,S may prove to be a valuable addition to malaria control efforts. However, the future development of more efficacious and long-lasting vaccines is likely to be needed to achieve elimination from many countries and regions. While the licensure of RTS,S will impact the way vaccine trials are conducted, testing second generation vaccines will remain feasible and achievable [103]. Combination vaccines appear crucial to achieving long-term objectives and there is still much to be done to prioritise candidates and combinations, and advance the most promising candidates into phase 2 trials.

### Competing interests

The authors declare that they have no competing interests.

### Acknowledgements

Funding was provided by the National Health and Medical Research Council of Australia (Senior Research Fellowship to JGB) and the Australia Research Council (Future Fellowship to FJIF). The Burnet Institute is supported by the NHMRC Independent Research Institutes Infrastructure Support Scheme, and a Victoria State Government Operational Infrastructure Support grant.

## The role of vector control in malaria eradication – opportunities and threats

**Fig. 12:**
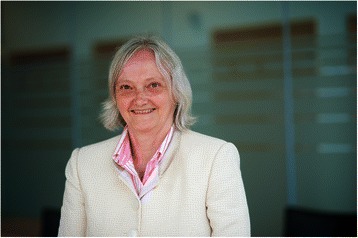
Janet Hemingway is Director of Liverpool School of Tropical Medicine and Professor of Insect Molecular Biology. She initially trained as a geneticist, and has 30 years of experience working on the biochemistry and molecular biology of specific enzyme systems associated with xenobiotic resistance. In recognition of her contributions to Tropical Medicine she is an Honorary Life Fellow of the Royal College of Physicians, the Royal Entomological Society and the American Academy of Microbiology, an elected Fellow of The Royal Society, an overseas Fellow of the National Academy of Sciences, USA, a Commander of the British Empire (CBE), and an elected Fellow of the Academy of Medical Sciences

Janet Hemingway (Fig. 12)

Effective prevention and control of insect borne diseases, such as malaria, in theory result from the integrated vector management (IVM) of the insects that transmit the parasites. In practice, there are two insecticide–based interventions, long-lasting insecticide impregnated bednets (LLINs) and indoor residual spraying (IRS) with proven efficacy at scale, as well as other interventions such as larval source management with recommendations for more limited use. Most of the recent documented reduction in transmission of malaria over the last decade can be attributed to massive scale up efforts in LLIN distribution and increased IRS, the latter predominantly driven by the US-backed Presidents Malaria Initiative (PMI) [104]. If we are to maintain this level of progress and transition from control to attempting to eradicate malaria, it is evident that there will need to be further scaling up of vector control, a recommendation that will be reflected in the updated Global Malaria Action Plan to be launched in 2015, projecting targets out to 2035. However, rapidly increasing levels of insecticide resistance, particularly in the two major African malaria vectors *Anopheles funestus* and *A. Gambiae* [105, 106], may seriously impact on these control interventions. Pyrethroids are the only class of insecticides recommended for LLINs and two thirds of IRS currently uses the same class. This over reliance on one insecticide class has resulted in a dramatic shift from almost no pyrethroid resistance in African vectors in the 1990s, to low level (~10-fold) resistance in the early 2000s in both vectors, to many reported cases today of >100- or >1,000-fold resistance [107].

Efforts are in place to address this problem. A product development partnership, the Innovative Vector Control (IVCC), was established in 2005 to stimulate and work with industry to develop novel public health-specific insecticides. There is now a healthy pipeline of new chemistries screened in partnership with all the major agrochemical companies, with a realistic expectation that we can bring three new insecticide classes to market before 2025. It is essential, before these new chemistries reach the market, that we have international agreement on and adoption of good insecticide resistance management programmes. A start has been made on this with the publication in 2013 of the Global Plan for Insecticide Resistance Management in disease vectors (GPIRM). It is, however, apparent that country programmes, non-governmental organisations (NGOs) and donor agencies are all struggling with the practical implementation of the principles of GPIRM, particularly where insecticide choice is already compromised by high levels of resistance to multiple classes of insecticide and shifts away from simple pyrethroid-based interventions incur significantly increased cost. Major collaborative work needs to be undertaken in this area, defining, implementing and supporting evidence-based best practice.

Extending the toolbox of effective scalable vector control interventions is a high priority. Demonstration of efficacy needs to be streamlined, so that we can dramatically truncate the 20+ years that it took to generate the evidence base for scaling of LLIN distribution. Several programmes have been established to define and test novel insect vector control paradigms. These include the use of spatial repellents [108], Wolbachia-based microbial control of pathogens in adult mosquitoes [109] and genetic manipulation of insect vector populations [110]. There are a number of large scale funding schemes supporting these activities and an international panel, the Vector Control Advisory Group, has been convened by WHO to work with innovators to develop and assess the evidence to support the mainstream introduction of promising technologies.

While these new interventions hold promise and all are entering small scale field trials, there is still a significant body of evidence required to assess whether any of these, in isolation or in combination with other well established interventions, can be scaled up to impact on transmission rates. Not all will be appropriate for malaria control; the Wolbachia approach, being applicable only for Culicine mosquitoes may, however, allow us to bring much needed new approaches to dengue control. Genetic manipulation approaches are starting to transition from the laboratory to proof of concept small scale field studies, allowing initial assessments of logistics, ease of application in resource poor settings, cost and public acceptability. The results of these trials will need to be independently scrutinised to determine whether the technologies are as yet sufficiently robust to warrant transitioning into large scale epidemiological impact trials. As novel public health insecticides and new technologies that go beyond the use of conventional insecticides start to emerge, an integrated vector management approach that utilises all the tools in the vector control toolbox will finally be possible to support the eradication effort.

### Competing interests

The author declares that she has no competing interests.

## Capacity building and leadership in malaria endemic countries

**Fig. 13:**
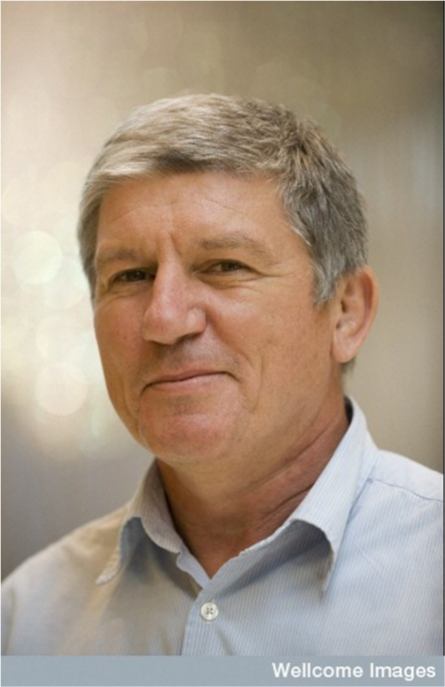
Kevin Marsh is a senior advisor at the African Academy of Sciences and professor of tropical medicine at the University of Oxford. Kevin has a particular interest in developing and strengthening research capacity and scientific leadership in Africa and is currently supporting the development of a new platform for the acceleration of science in Africa through the African Academy of Sciences. He is chair of the WHO Malaria Policy Advisory Committee and is a member of a number of international advisory committees relating to malaria and to global health research. Image reproduced with permission from the Wellcome Trust

**Fig. 14:**
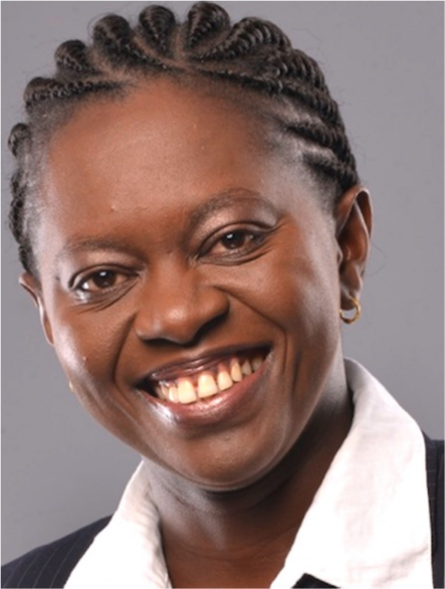
Faith Osier is a Wellcome Trust Clinical Research Fellow and an MRC/DfID African Research Leader based at the KEMRI-CGMR-C in Kilifi, Kenya where she leads a group of African scientists. She is a Visiting Professor of Immunology at the University of Oxford and the Secretary General of the Federation of African Immunological Societies (FAIS)

Kevin Marsh (Fig. 13) and Faith Osier (Fig. 14)

The last 15 years have seen a remarkable galvanisation of national and international efforts in malaria control leading to striking reductions in malaria transmission, case burden and mortality [3]. There is now an international consensus that local malaria elimination leading to global eradication must be the explicit long term aim of national and global efforts. Achieving this will require major investments to strengthen capacity for both research and the programmatic ability to deliver interventions. Whilst there are important ways in which requirements for control differ from those for elimination [111], we should remember control is a prerequisite for elimination and at the moment is the highest global priority. While the capacity required for both research and programme implementation includes human resources, infrastructure and management, we believe that an absolute requirement is a major increase in long term investment to massively increase the size and skill sets of professional cadres in malaria endemic countries and an emphasis on building outstanding leadership.

One of the most important lessons from the global polio eradication programme was the importance of maintaining strong investment in research, even when the tools are thought to be at hand. In the case of malaria, this lesson is even more pertinent, as most experts believe that even with maximal application of currently available tools, it will not be possible to eliminate malaria in many settings. Between 2008 and 2010 a wide ranging international consultative effort defined priorities in all areas of basic and enabling research necessary for eradication [2]. Whilst it is important to identify key innovations and interventions, an overarching need is to increase the research capacity within endemic countries [112]. Malaria-specific research cannot be separated from the wider issues of harnessing science to tackle developmental goals and it is important to realise the scale of the challenges. Major industrialised economies typically have researcher ratios in the order of 4,000 per million of population [10]. Some malaria endemic countries particularly in South America and Asia are developing a strong research base but for most African endemic countries, which represent 90 % of the world’s malaria burden, the figures are often of the order of less than 50 per million, i.e. two log orders less than in resource rich countries. Over the last fifteen years there has been substantial focused investment in building scientific capacity for malaria research by a number of international funders including the Bill and Melinda Gates Foundation, the US National Institutes of Health (NIH), the Wellcome Trust, and the European & Developing Countries Clinical Trials Partnership (EDCTP) among others. National investments in some malaria endemic countries also show signs of improvement but much increased sustained investment is still needed.

Malaria control and subsequent elimination depend absolutely on having well-organised, adequately staffed and highly skilled workforces. Just as malaria specific research cannot be considered in isolation, so malaria control cannot be separated from the broader issues facing health service delivery. Again, the picture is often one of a worrying underinvestment, with many of the highest burden countries remaining well below any acceptable minimum level [113, 114]. For instance, Africa has 24 % of the global disease burden but only 3 % of the global health workforce. This imbalance is reflected in the key cadres necessary for effective malaria control. Vector control activities account for around 60 % of global expenditure on malaria control but a recent analysis points to the massive gap in capacity on the ground [114]. A similar situation applies across other key areas of expertise and is consistently identified in Malaria Country Programme Reviews across all WHO regions (K Mendis and M Warsame personal communication). As with the research gap, there are hopeful signs, especially in terms of regional political commitment through organisations such as the Asia Pacific Leaders Malaria Alliance (APLMA) and the African Leaders Malaria Alliance (ALMA), but investment currently runs far below requirements.

Whilst investment is needed in all domains and for all cadres, the single most important factor for developing the necessary capacity in both research and programmatic capacity is the fostering of strong leadership. As well as formulating new strategies, effective leaders inspire others, advocate for political support and mobilise the funding necessary to drive whole fields of endeavour. It is often tempting to focus on technical fixes and the possibility of circumventing deficits by time limited solutions but whilst external financing and expertise will continue to play a critical role, it is hard to see how sustained control and elimination will be achieved in any country that has not been through the process of building its own capacity and leadership. Developing influential leadership in any area takes time, typically a minimum of 10 to 15 years from primary qualification. There is now considerable emerging experience of how to do this [115] and the main limitation is funding. That being the case, and malaria eradication being a long term enterprise, what is needed is a serious large scale investment by international, regional and national stakeholders in building the leaders of the future for malaria research, control and eradication.

### Competing interests

The authors declare that they have no competing interests.

## Box 1 Consequences of resistance

Greater economic impact

Delay in the initial therapeutic response

Recrudescent infections

More anaemia following repeated infections

Increased gametocyte carriage and transmission

Increased incidence of malaria

A greater frequency and severity of epidemics

Increased mortality
